# Did Resilience and Socioeconomic Status Predict Older Adults’ Finding a Silver Lining in COVID?

**DOI:** 10.1093/geroni/igad058

**Published:** 2023-06-21

**Authors:** Jocelyn Wilder, Diane S Lauderdale, Louise Hawkley

**Affiliations:** NORC at the University of Chicago, Chicago, Illinois, USA; Department of Public Health Sciences, The University of Chicago, Chicago, Illinois, USA; NORC at the University of Chicago, Chicago, Illinois, USA

**Keywords:** COVID-19 pandemic, Fundamental cause theory, Older adults, Resilience

## Abstract

**Background and Objectives:**

The coronavirus disease 2019 (COVID-19) pandemic stretched our limits—physically, mentally, and economically. However, some older adults report that it led to positive changes. This study aims to understand whether prepandemic resilience, education, or income predicted older adults’ subsequent likelihood of reporting positive changes in their lives during the pandemic.

**Research Design and Methods:**

We use data from the National Social Life, Heath, and Aging Project, an ongoing panel study with a COVID-19 ancillary supplement (*N* = 2,650).

**Results:**

The study results aligned with the fundamental cause theory. In demographically adjusted models including resilience, education, and income, as well as the effect of the pandemic on employment and a COVID-disruption score, the odds of reporting any positive change were 2.6 times higher for those with an associate degree (*p* < .01) and 4.7 times higher for those with a bachelor’s or higher (*p <* .001), compared to those without a high school degree. In contrast, neither resilience nor income was significantly associated with endorsing a positive change. We also categorize specific changes thematically coded from open-ended responses and examine their demographic distributions. Categories include spirituality, home organization, hygiene practices, and increased quality time with others.

**Discussion and Implications:**

These findings show that older adults with more education could navigate COVID-19 challenges in a way that improved their perspectives on at least one aspect of their lives.

**Translational Significance:** The study, which used panel data from the National Social Life, Health, and Aging Project both prepandemic and during the coronavirus disease 2019 pandemic, highlights the relevance of the fundamental cause theory in a broader context. The results supply valuable insights into the role of adaptable resources in achieving positive outcomes during challenging circumstances. These findings have potential implications for understanding the link between resilience and the fundamental cause theory, and they contribute to the ongoing conversation about identifying assets that can account for favorable outcomes in socioeconomically disadvantaged populations.

The coronavirus disease 2019 (COVID-19) pandemic (pandemic) exposed how structural and societal inequities in health and material circumstances could lead to widening health disparities. Older adults specifically may have experienced increased vulnerability across multiple domains, including physical health, mental health, and financial stability during the pandemic (Bui et al., 2021; Koma et al., 2020; Lesser & Nienhuis, 2020; Li & Mutchler, 2020; Vahia et al., 2020; Whitehead & Torossian, 2021). However, some older adults have found silver linings despite pandemic-related vulnerabilities. For instance, whereas social distancing and stay-at-home mandates restricted movement and social interactions, some found that these restrictions gave way to increased or more meaningful time with loved ones, home improvements, greater reflection, and new hobbies. How is it that some older adults were able to create or identify positive impacts within the pandemic? We examined two possible facilitators, socioeconomic status (SES), measured by prepandemic household income (income) and educational attainment, and trait resilience (resilience), also measured prepandemic, which may enable older adults to overcome the challenges of the COVID-19 pandemic and experience positive changes in their lives.

Few studies have assessed the association between prepandemic measures (educational attainment, household income, or resilience) and outcomes during the pandemic. One study found that prepandemic parental educational attainment was associated with parental and child well-being, and higher educational attainment was related to less negative economic impact during the early stages of the pandemic (Porter et al., 2021). Choi et al. (2023) found that higher resilience (measured prior to January 2020) was associated with lower distress and higher positive emotional well-being. This study’s limitation is that the study population of older female nurses and health care workers may not represent the general population during the pandemic.

Fundamental cause theory (FCT) explains why associations between SES and health disparities due to structural inequities endure even as health problems and health care change. FCT posits that personal resources such as money, knowledge/education, power, prestige, or social connections can be flexibly used to attain health-related knowledge, access helpful or needed services, or purchase preventative and curative technologies. Individuals embedded in social contexts (e.g., workplace, neighborhood, and peer networks) contribute to differential exposure to health threats and self-protection opportunities (Link & Phelan, 1995). FCT may explain how people can maneuver new challenges, such as the COVID-19 pandemic. For example, resources such as higher income may increase one’s ability to take advantage of remote means of conducting everyday activities (e.g., home delivery of groceries). The pandemic altered the social context for many people. Social distancing, prolonged illness (long COVID), or losing a friend or family member may have affected peer and familial networks through increased engagement with or shrinkage of contacts (Bertogg & Koos, 2022). Additionally, residential relocation may have occurred in response to social distancing guidelines (e.g., to create bubbles/pods) or out of financial or caregiving necessities. There are additional ways that more resources could have affected the pandemic experience, such as providing opportunities for virtual social interactions or goods for new hobbies.

Whereas FCT posits that the ability to deploy resources flexibly helps people weather adversity, the psychological construct of trait resilience posits that individuals differ in their tendency to withstand, recover, and bounce back from stress and adversity (Hawkley et al., 2021). As a trait, resilience draws on self-esteem, self-efficacy, critical thinking skills, optimism, and purpose, attributes that help individuals maintain a positive mindset, reframe a challenge, or seek social support. Higher trait resilience increases individuals’ likelihood of finding the positive and making positive changes during adversity (Fuller & Huseth-Zosel, 2021). The extent to which prepandemic education and income are related to resilience among older adults has yet to be extensively examined. Resilience may be independently related to endorsing a positive change or may fall downstream as a consequence of educational attainment and income. Resilience may be built over a person’s life through encountering challenging but manageable situations, thus preparing individuals for later stressors (Ong & Leger, 2022). Earlier stressors, such as those that occurred during education, while in the workforce, or in family or social contexts before the pandemic, may have prepared older adults better for the challenges of the pandemic (Phillips et al., 2016; Shing et al., 2016). To the extent that resilience facilitates the identification and endorsement of positive change in the face of stress, it may mediate the relationship between stressors and life satisfaction (Kim et al., 2017; Seligowski et al., 2015). This study explores (a) the relationship between socioeconomic resources and resilience and (b) the relationships of socioeconomic resources and resilience with endorsements of positive change during the pandemic for older adults.

## Method

### Sample

The COVID supplemental survey sample includes 4,852 previous National Social Life Health and Aging Project (NSHAP) respondents (2,531 Cohort 1 birth years 1920 to 1947 and 2,321 Cohort 2 birth years 1948 to 1965). The sample did not include 96 cases determined to be deceased or hard refusals before the survey and 569 cohort 2 nonrespondent cases. Data were collected between September 14, 2020, and January 27, 2021, using web, phone, and paper-and-pencil surveys. The end of this period corresponds with the beginning of the general availability of vaccines. Responses were received from 2,672 individuals, resulting in a conditional response rate of 60.9% for cohort 1, 56.2% for cohort 2, and 58.1% for both cohorts combined. Because spouses were included in NSHAP, and some were younger than the target ages, the study sample was limited to 55 years and older to capture only those in the target NSHAP age range at the time of recruitment; the final sample contained 2,465 respondents.

### Dependent Variable

The dependent variable was the endorsement of any positive changes during the pandemic. Respondents were asked, “Has the COVID-19 pandemic led to any positive changes in your life?” with “yes” and “no” response options. This binary response is the primary outcome variable. “Yes,” responses were followed up with a request to share an example of a positive change in a free-text box. An iterative collaborative inductive process with an external researcher used thematic analysis to examine the open-ended responses. Open-ended responses were read, frequently appearing words were identified and cataloged, and a coding lexicon was developed to generate themes. The identified themes were spirituality, social relationships, health behaviors, homemaking, hygiene, financial management, stress management, and hobbies.

Responses that included “church, bible, pray, worship, meditation, reflection, spiritual, or God” were categorized initially as religion or self-reflection and later condensed into one theme representing *spirituality*. Responses that included “getting to know, rekindled, communication, closer, quality, and time with family, friends, relatives, or children” were grouped as relating to quality time and family and later condensed into themes representing *social relationships*. Responses that included “health, diet, quit drinking or drinking less, quit smoking, or smoking less, weight loss, exercise, physical activity, and/or walking” were grouped as relating to better health, diet, or exercise (such as walking or tennis) and were later condensed to *health behaviors*. Responses that included “cleaning, organizing, home projects, paring down, remodeling, renovation, or redecorating” were grouped as home organization, cleaning, or remodeling and were later condensed to *homemaking*. The hygiene theme included responses such as “social distancing, disinfecting, sanitizing, staying away from others, masking, or wearing gloves.” The theme of *financial management* included responses such as “money, finances, saving money, spending, or income. *Stress management* included responses such as “social anxiety, calming, stress, balance, or slowed down.” *Hobbies* included responses such as “playing music, bridge, sewing, knitting, quilting, painting, reading, writing, or birdwatching.” Respondents who responded yes to the initial question, “Has the COVID-19 pandemic led to any positive changes in your life?” but did not write in a specific positive change were classified as “other” along with infrequent or difficult-to-classify responses such as “no tv” or “no obnoxious neighbors.” Open-ended responses were not mutually exclusive and were assigned to multiple themes. For example, responses such as “exercise more, bonded more with family, organized house better” were classified as health behaviors, social relationships, and homemaking. Some unusual examples of individual responses and their categorization included: “I gained so much weight that I am finally eligible for weight loss surgery which I am working with doctors on getting” (health behaviors); “confined no travel no run girls [commercial sex workers]” (other), and “allayed my social anxiety I am a true introvert” (stress management).

### Independent Variables

To examine FCT, we used measures of educational attainment and household income reported in 2015–2016. Educational attainment was categorized into four levels: non-high school graduates, high school (HS) graduates/General Education Development (GED), associate degree/vocational degree, and college/graduate degree. Household income was grouped into four categories, <25,0000, 25,000–49,999, 50,000–99,999, and ≥100,000. Resilience was derived from the 4-item trait resilience measure (Hawkley et al., 2021). The four items: “I bounce back quickly after hard times,” “I am an energetic person,” “I take things in stride,” and “I can do just about anything I really set my mind to” were each rated on a 4-point scale: never (0), some of the time (1), usually (2), and always (3). Responses were summed to create a resilience score ranging from 0 to 12, with higher scores representing greater resilience.

### Covariates

Additional sociodemographic measures from the 2015–2016 NSHAP survey were respondents’ sex, age, and race/ethnicity (categorized as Hispanic, non-Hispanic (NH) Black, NH Other, and NH White). Those responding as Native American/Alaskan Native (0.74%), Asian/Pacific Islander (2.0%), or Other were categorized as “NH Other” due to the small sample size. Current marital status and whether the pandemic affected current employment (categorized as “No, not working at the start of the pandemic,” “No,” and “Yes”) were reported in the COVID-19 supplemental survey. A COVID-19 event score captured disrupting events during the pandemic that may be associated with the level of challenge that the pandemic represented for individual respondents. The COVID-19 event score was summed across five items: “Live alone” (no/0 vs yes/1), “Know someone who died from COVID-19” (no/0 vs yes/1), “Did anyone move in with you due to the pandemic?” (no/0 vs yes/1), “Did you change where you lived due to the pandemic?” (no/0 vs yes/1) and one derived item about how the pandemic affected income. Responses to the latter question were categorized as “Same as before/Better off” (0) versus “Worse off” (1). “Lives alone” was included because of the greater potential effect of social distancing requirements and guidelines on quality of life. The COVID-19 event score ranged from 0 to 5, with higher scores representing greater disruption.

### Analysis

All analyses used STATA 17 (Stata Corporation LP, College Station, TX). Correlations explored the relationship between resilience and socioeconomic measures (educational attainment and household income). Resilience was modeled as a dependent variable in linear regressions to examine its association with socioeconomic measures (education and household income) separately and together. Chi-square tests assessed group differences in endorsing a positive change as a function of independent variables and covariates. Independent variables were modeled separately and together to assess their relationships with the endorsement of any positive change. Logistic regression models were adjusted for gender, age, race/ethnicity, marital status, the effect of the pandemic on work, and COVID disruption score. Descriptive statistics and all models were survey-weighted (using weights derived from the COVID-19 supplemental survey by the reciprocal probability of selection probability) and adjusted for nonresponse based on age and urbanicity.

## Results

Table 1 describes the characteristics of the sample. The mean age was 68 years. More than half of the sample was female (55%), and 71% of the sample’s educational attainment was associate/vocational degree or higher. Approximately 62% of respondents reported household incomes above or equal to $50,000. Most of the sample had a resilience score of 7 or higher (mean resilience score = 8.0, 95% CI: 7.9–8.1). About half the sample was not in the workforce at the pandemic’s beginning.

**Table 1.** Descriptive Statistics of the National Social Life, Heath, and Aging Project COVID-19 Sample (*n* = 2,465), Age 55 Years and Older (Weighted)

Variable	Percent or Mean (95% CI)	Positive change (%)^a^		
		No	Yes	*p* Value
Overall		62.5	37.5	
Gender (%)				
Female	55.2 (53.4, 57.0)	58.4 (55.5, 61.3)	41.6 (38.7, 44.5)	*p <* .001
Male	44.8 (43.0, 46.6)	67.5 (63.4, 71.4)	32.5 (28.6, 36.6)	
Age in years (Mean)	68.0 (67.4, 68.6)	68.9 (68.2, 69.6)	66.1 (65.4, 66.8)	*p*_trend_ < .001
55–64 years	43.1 (40.0, 46.3)	56.3 (52.6, 60.0)	43.7 (40.0, 47.4)	
65–79 years	44.0 (41.3, 46.8)	65.0 (61.1, 68.6)	35.0 (31.4, 38.9)	
≥80 years	12.8 (11.2, 14.6)	75.7 (70.6, 80.1)	24.3 (19.9, 29.4)	
Race/ethnicity (%)				
Hispanic	6.7 (5.3, 8.6)	68.4 (59.4, 76.1)	31.6 (23.9, 40.6)	*p <* .001
NH-Black^b^	10.4 (8.1, 13.4)	47.3 (40.2, 54.5)	52.7 (45.5, 59.8)	
NH-Other^c^	3.5 (2.6, 4.7)	51.0 (36.6, 65.3)	49.0 (34.7, 63.4)	
NH-White	79.3 (75.7, 82.6)	64.4 (61.2, 67.4)	35.6 (32.6, 38.8)	
Marital status (%)				*p* = .18
Living with partner	4.1 (2.9, 5.6)	67.9 (55.9, 77.9)	32.1 (22.1, 44.1)	
Married	66.3 (63.3, 69.1)	60.3 (56.5, 64.1)	39.7 (35.9, 43.5)	
Never married	4.8 (3.8, 6.1)	64.5 (54.9, 73.0)	35.5 (27.0, 45.1)	
Separated/divorced	13.2 (11.4, 15.2)	64.8 (58.4, 70.7)	35.2 (29.3, 41.6)	
Widowed	11.6 (10.5, 12.9)	68.2 (61.6, 74.2)	3.8 (25.8, 38.4)	
Educational attainment (%)				*p*_trend_ < .001
No HS diploma	7.4 (6.0, 9.0)	81.5 (73.4, 87.6)	18.5 (12.4, 26.6)	
HS diploma/GED	21.2 (18.7, 24.0)	73.6 (68.9, 77.8)	26.4 (22.2, 31.1)	
Associates/vocational	36.1 (33.4, 38.9)	64.5 (60.6, 68.2)	35.5 (31.8, 39.4)	
Bachelors/graduate	35.3 (32.0, 38.7)	50.5 (45.8, 55.2)	49.5 (44.8, 54.2)	
Household income (%)^d^				*p*_trend_ < .001
<25,000	17.2 (14.8, 19.8)	69.9 (63.4, 75.2)	30.4 (24.8, 36.6)	
25,000–49,999	20.4 (18.1, 23.0)	65.9 (61.2, 70.3)	34.1 (29.7, 38.8)	
50,000–99,999	31.3 (28.9, 33.8)	66.4 (62.0, 70.5)	33.6 (29.5, 38.0)	
≥100,000	31.0 (27.9, 34.3)	52.3 (47.2, 57.2)	47.7 (42.8, 52.8)	
Resilience score (Mean)^e^	8.0 (7.9, 8.1)	8.0 (7.8, 8.1)	8.2 (8.0, 8.4)	*p*_trend_ = .020
1–3	1.5 (1.0, 2.2)	90.3 (67.7, 97.6)	9.7 (2.4, 32.3)	
4–6	17.4 (15.3, 19.7)	67.5 (61.6, 73.0)	32.5 (27.0, 38.4)	
7–9	60.3 (57.7, 62.7)	60.8 (57.2, 64.3)	39.2 (35.7, 42.8)	
10–12	20.8 (18.8, 23.0)	62.9 (56.6, 68.8)	37.1 (31.2, 43.4)	
Work affected by COVID-19 (%)				*p <* .001
Not in work force	48.2 (45.7, 50.7)	68.1 (64.9, 71.2)	32.0 (28.8, 35.1)	
No	20.3 (18.2, 22.6)	65.5 (59.6, 71.0)	34.5 (29.0, 40.4)	
Yes	31.2 (29.2, 33.9)	52.6 (48.4, 56.8)	47.4 (43.2, 51.6)	
COVID disruption score (%)^f^				*p* = .424
0	54.6 (51.7, 57.5)	63.5 (59.8, 67.2)	36.5 (32.8, 40.2)	
1	35.8 (33.2, 38.5)	61.9 (58.2, 65.6)	38.1 (34.4, 41.8)	
≥2	9.5 (8.0, 11.3)	58.2 (50.2, 65.8)	41.8 (34.2, 49.8)	

*Notes*: GED = General Education Development; HS = high school; NH = non-Hispanic.

^a^Of the respondents asked, “Has the COVID-19 pandemic led to any positive change in your life” 162 are missing responses.

^b^Survey race question did not distinguish between African American and Black.

^c^Races categorized as Other were those who responded Native American/Alaskan Native (0.74%), Asian/Pacific Islander (2.0%), and Other (7.6%).

^d^Household income was derived from unfolding brackets; those responding, “don’t know/refused” were asked the specific income question, “Would you say the income of your household was more or less than $50,000? Those responding less were asked, “Would you say the income of your household is more than $25,000 or less than $25,000?” and those responding more were, “Would you say the income of your household is more than $100,000 or less than $100,000?”

^e^Resilience score was calculated from the 4-item trait resilience measure. Responses were summed to create a total resilience score ranging from 0 to 12, with higher scores representing greater resilience.

^f^The COVID disruption score was calculated by summing five dichotomous items: living alone, COVID-19-related deaths among friends/family, residential relocation of self, others moving in due to the pandemic, and pandemic effect on income. The disruption score ranged from 0 to 5, with higher scores representing greater disruption.

**Figure 1.**
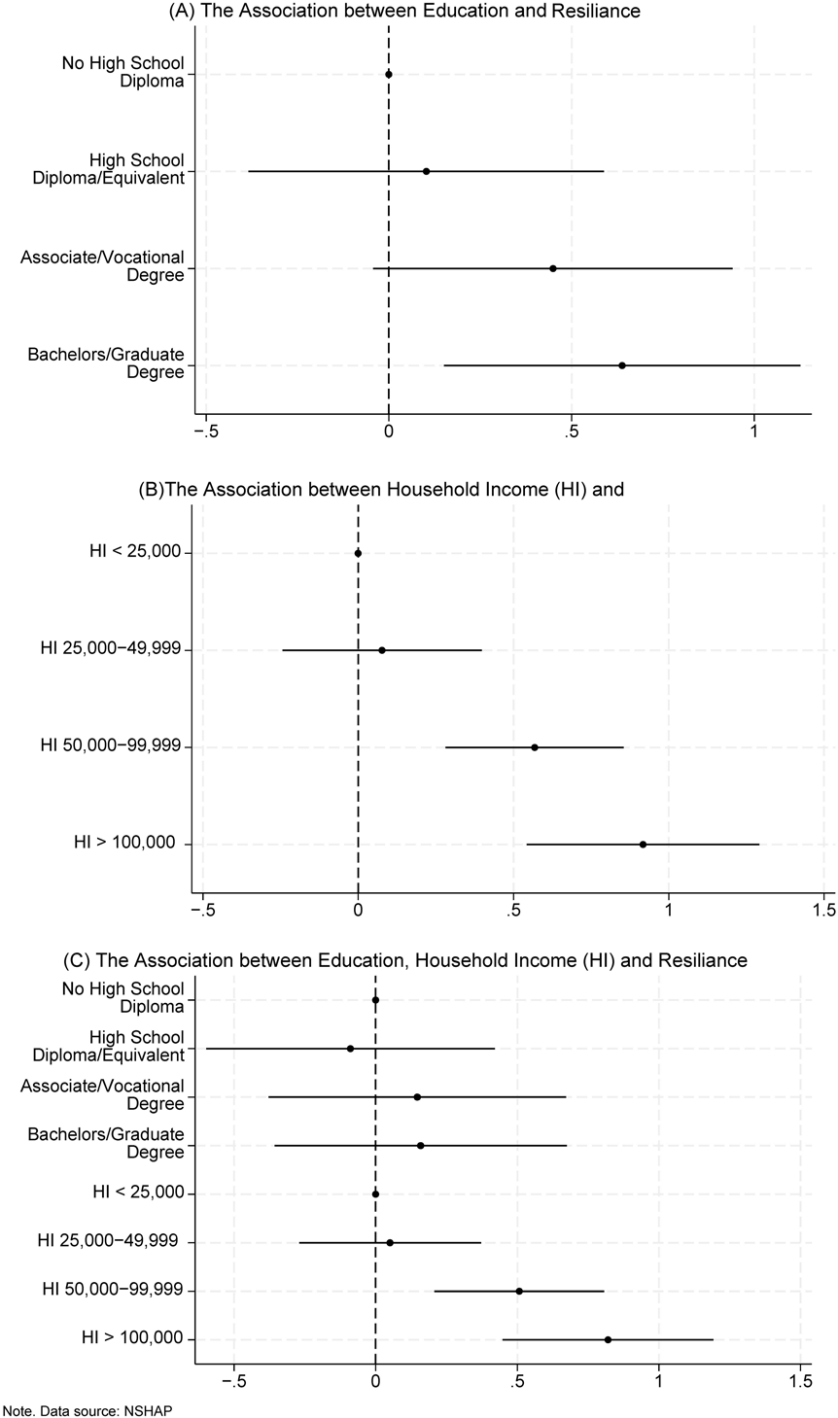
The association^a^ between education^b^, household income (HI)^c^, and resilience^d^ in the National Social Life, Heath, and Aging Project COVID-19 sample, aged 55 years and older (weighted). (**A**) Shows how educational attainment is associated with resilience. Attainment of a bachelor’s degree or higher was associated with higher resilience scores than those without a high school diploma. There were no statistically significant differences in resilience between those with a high school diploma, those with associate/vocational degrees, and those without a high school diploma. (**B**) Shows how HI is associated with resilience. Household incomes of $50,000–$99,999 and $100,000 or more were associated with higher resilience scores than HIs less than $25,000. (**C**) Shows how educational attainment and HI are associated with resilience. Higher HI was statistically significantly associated with higher resilience scores, even after controlling for educational attainment. However, the association between educational attainment and resilience score was not statistically significant after controlling for HI. ^a^The x-axis shows the unadjusted coefficients and 95% confidence intervals (CI) of resilience, which were estimated from a linear regression model with educational attainment as the independent variable (1A), HI as the independent variable (1B), and educational attainment and HI as independent variables (1C). ^b^Educational attainment was categorized into four levels: non-high school (HS) graduates, HS graduates/GED, associate degree/vocational degree, and bachelors/graduate degree. ^c^Household income was derived from unfolding brackets; responses such as “do not know/refused” were asked the specific income question “Would you say the income of your household was more or less than $50,000? Those responding less were asked, “Would you say the income of your household is more than $25,000 or less than $25,000?” and those responding more were, “Would you say the income of your household is more than $100,000 or less than $100,000?.” ^d^Resilience score was calculated from the 4-item trait resilience measure. Responses were summed to create a total resilience score ranging from 0 to 12, with higher scores representing greater resilience.

Resilience had significant but weak positive relationships with educational attainment (*r* = 0.10) and household income (*r* = 0.16; Supplementary Table 1). A bachelor’s degree or higher was associated with higher resilience scores than those without a HS diploma (β = 0.64, 95% CI: 0.15–1.1, *p* = .011). There were no statistically significant differences in resilience between those with HS and those with associate/vocational degrees compared to those without HS diplomas (Figure 1A). Household incomes of $50,000–$99,999 (β = 0.57, 95% CI: 0.28–0.86, *p* < .001) and $100,000 or more (β = 0.92 95% CI: 0.52–1.3, *p* < .001) were associated with higher resilience scores than household incomes less than $25,000 (Figure 1B). In an adjusted model containing household income and educational attainment, higher household income remained significantly associated with higher resilience scores. However, the association between educational attainment and resilience score was attenuated and no longer significant (Figure 1C).

Thirty-eight percent of respondents endorsed any positive change during the pandemic (Table 1). Endorsement of positive change increased monotonically with educational attainment; approximately 50% of those with college/graduate degrees endorsed a positive change compared to 36% with associates/vocational degrees, 26% of HS graduates, and 19% with no HS diploma. The pattern suggested more of a threshold effect for income. Forty-eight percent of respondents with household incomes greater than $100,000 endorsed any positive change compared to 34% for incomes 50K–99.9K, 34% for incomes 25K–49.9K, and 30% for incomes <25K.

Among respondents describing a specific positive change, the top three themes identified were social relationships (20%), spirituality (15%), and health (12%; Table 2). Forty-one percent of respondents endorsing a positive change were classified in the “other” theme; most did not write in a specific change, and fewer specified a change that did not align with the defined themes. Those who did not describe their positive change differed little by education, income, or resilience from those who did describe their change (Supplementary Table 2).

**Table 2.** Adjusted Odds Ratios for the Association Between Education, Income, Resilience, and Endorsement of a Positive Change (Weighted)

Variable	Unadjusted models	Model 1-education^a^	Model 2-income^b^	Model 3-resiliance^c^	Model 4-final model^d^
	OR (95% CI)	OR (95% CI)	OR (95% CI)	OR (95% CI)	OR (95% CI)
Educational attainment	*p*_trend_ < .001	*p*_trend_ < .001			*p*_trend_ *<* .001
No HS diploma	Reference	Reference			Reference
HS diploma/GED	1.58 (0.95, 2.63)	1.72 (0.96, 3.05)			1.73 (0.88, 3.39)
Associates/vocational	2.43 (1.52, 3.90)***	2.53 (1.45, 4.40)**			2.62 (1.34, 5.10)**
Bachelors/graduate	4.32 (2.68, 6.96)***	4.95 (2.81, 8.74)***			4.74 (2.42, 9.27)***
Household incomee	*p*_trend_ < .001		*p*_trend_ < .001		*p*_trend_ = .84
<25,000	Reference		Reference		Reference
25,000–49,999	1.18 (0.86, 1.63)		1.18 (0.83, 1.70)		0.90 (0.59, 1.37)
50,000–99,999	1.16 (0.85, 1.59)		1.17 (0.82, 1.68)		0.78 (0.52, 1.18)
≥100,000	2.09 (1.50, 2.92)***		2.01 (1.38, 2.93)***		1.03 (0.67, 1.59)
Resilience score^f^	1.05 (0.99, 1.11)			1.07 (1.01, 1.14)*	1.05 (0.98, 1.11)
Age (continuous)	0.97 (0.96, 0.98)***	0.98 (0.96, 0.99)**	0.98 (0.97, 0.99)*	0.98 (0.96, 0.99)**	0.98 (0.96, 0.99)*
Gender					
Female	Reference	Reference	Reference	Reference	Reference
Male	0.68 (0.55, 0.82)	0.61 (0.48, 0.76)***	0.61 (0.48, 0.77)***	0.65 (0.52, 0.82)***	0.58 (0.45, 0.74)***
Race/ethnicity					
NH White	Reference	Reference	Reference	Reference	Reference
Hispanic/Latinx	0.84 (0.57, 1.23)	0.94 (0.65, 1.37)	0.81 (0.55, 1.20)	0.80 (0.53, 1.21)	1.01 (0.66, 1.55)
NH Black^g^	2.01 (1.4, 2.81)***	2.36 (1.70, 3.27)***	2.21 (1.59, 3.07)**	2.10 (1.43, 3.06)***	2.50 (1.73, 3.60)***
NH Other^h^	1.73 (0.93, 3.22)	1.42 (0.70, 2.90)	1.66 (0.80, 3.46)	1.60 (0.74, 3.44)	1.47 (0.63, 3.44)
Marital status					
Married	Reference	Reference	Reference	Reference	Reference
Living with partner	0.72 (0.42, 1.24)	0.67 (0.38, 1.17)	0.71 (0.41, 1.21)	0.44 (0.21, 0.94)*	0.50 (0.24, 1.08)
Never married	0.84 (0.56, 1.27)	0.62 (0.38, 1.01)	0.72 (0.43, 1.19)	0.70 (0.41, 1.20)	0.61 (0.34, 1.08)
Separated/divorced	0.83 (0.59, 1.16)	0.66 (0.44, 1.01)	0.78 (0.50, 1.21)	0.60 (0.39, 0.93)*	0.68 (0.43, 1.06)
Widowed	0.71 (0.51, 0.98)*	0.92 (0.60, 1.41)	0.84 (0.55, 1.28)	0.87 (0.58, 1.29)	0.96 (0.61, 1.50)
Work affected by COVID-19^i^					
Not in work force	Reference	Reference	Reference	Reference	Reference
No	1.12 (0.84, 1.51)	1.02 (0.77, 1.38)	0.94 (0.71, 1.25)	0.95 (0.68, 1.32)	0.97 (0.69, 1.35)
Yes	1.92 (1.55, 2.34)	1.31 (1.01, 1.70)*	1.3 (0.99, 1.68)	1.52 (1.17, 1.98)**	1.3 (0.95, 1.66)
COVID disruption score^j^	1.12 (0.96, 1.30)	1.15 (0.95, 1.39)	1.1 (0.93, 1.39)	1.14 (0.92, 1.41)	1.1 (0.92, 1.37)

*Notes*: GED = General Education Development; HS = high school; NH = non-Hispanic.

^a^Model 1 is the main effect of education adjusted for age, sex, race/ethnicity, education, marital status, and COVID disruption score.

^b^Model 2 is the main effect of household income adjusted for age, sex, race/ethnicity, education, marital status, and COVID disruption score.

^c^Model 3 is the main effect of resilience adjusted for age, sex, race/ethnicity, education, marital status, and COVID disruption score.

^d^Model 4 is the full model containing education, household income, and resilience adjusted for age, sex, race/ethnicity, education, marital status, and COVID disruption score.

^e^Household income was derived from unfolding brackets; those responding, “don’t know/refused” were asked the specific income question, “Would you say the income of your household was more or less than $50,000? Those responding less were asked, “Would you say the income of your household is more than $25,000 or less than $25,000?” and those responding more were, “Would you say the income of your household is more than $100,000 or less than $100,000?”

^f^Resilience score was calculated from the 4-item trait resilience measure. Responses were summed to create a total resilience score ranging from 0 to 12, with higher scores representing greater resilience.

^g^National Social Life, Heath, and Aging Project’s race question did not distinguish between African American and Black.

^h^ Races categorized as Other were those who responded Native American/Alaskan Native (0.74%), Asian/Pacific Islander (2.0%), and Other (7.6%).

^i^Respondents were asked “Has your work been affected by the COVID-19 pandemic?” “Not working” capture those who were not working when the pandemic started.

^j^The COVID disruption score was calculated by summing five dichotomous items: living alone, COVID-19-related deaths among friends/family, residential relocation of self, others moving in due to the pandemic, and pandemic effect on income. The disruption score ranged from 0 to 5, with higher scores representing greater disruption.

*p < .05. **p < .01. ***p < .001.

Table 2 displays the unadjusted results for each covariate separately in the first column, followed by three models that examine the effect of each main exposure variable separately (educational attainment, household income, and resilience score). Model 1 through Model 3 examined the effects of education, income, and resilience separately on endorsing a positive change, with each model adjusted for demographics (age, gender, race/ethnicity, marital status), current employment status, and COVID disruption score. In these separate adjusted models, the positive trend of the SES measures (educational attainment [*p*_trend_ < .001] and household income [*p*_trend_ < .001]) with the endorsement of any positive change remained. Resilience scores (Model 3) were marginally associated with positive change (adj OR = 1.07; 95% CI: 1.01, 1.14).

In the fully adjusted model (Model 4) that included educational attainment, household income, and resilience, only educational attainment remained significantly associated with endorsing a positive change. There was a highly significant trend (*p <* .001) and a pattern of monotonically increasing odds with greater educational attainment: relative to no HS; the adjusted odds ratios were 1.73 (95% CI: 0.88, 3.40) for HS diploma/GED, 2.62 (95% CI: 1.34, 5.10) for associate/vocational degree, and 4.74 (95% CI: 2.42, 9.27) for college/graduate degrees. Increasing age, female gender, and Black race were significantly associated with positive change endorsement in Model 4.

Some categories of specific positive changes varied by respondents’ characteristics (Table 3). Younger respondents (55–64 years) had a higher occurrence of changes related to social relationships (25.4%) compared to older adults 65–79 years (19.4%) and 80-plus years (11.5%). NH Black respondents had a higher occurrence of changes related to spirituality (20.7%) and hygiene (16.8%) compared to Hispanic (18.9% and 11.3%), NH Other (18.9% and 8.1%), and NH White respondents (12.9% and 9.6%). In terms of educational attainment, respondents who did not complete HS had higher occurrences of changes related to hygiene (18%) than those with HS diplomas (15%), associates/vocational degrees (14%), and college/graduate degree (7%). Respondents with a college degree or higher reported a higher occurrence of changes related to health behavior (14%) than those with associate/vocational degrees (11%), HS diplomas (8%), and noncompletion of HS (3%). Respondents reporting household income ≥100,000 also reported higher occurrences of change related to health behaviors (15.4%) than those reporting lower household incomes. Those with higher resilience scores (>10) had higher occurrences of changes related to health behavior change (17.7%) compared to respondents with lower resilience scores.

**Table 3.** Prevalence of Specific Positive Change Among Respondents Endorsing a Positive Change^a^ by Selected Sociodemographic Characteristics (Unweighted *n* = 2,558)

Variable	Positive change (N)	Social relationships^b^	Spirituality^c^	Health behaviors^d^	Homemaking^e^	Hygiene^f^	Financial management^g^	Stress management^h^	Hobby^h^^,^^i^	Other^j^
Overall (*N*)	822	166	123	99	57	90	15	30	45	337
Percent		20.2	15.0	12.0	6.9	11.0	1.8	3.7	5.5	41.0
Gender										
Female (*n* =1,449)	515	20.0	17.9	12.4	6.6	11.3	2.3	4.5	5.8	38.6
Male (*n* = 1,109)	307	20.5	10.1	11.4	7.5	10.4	0.98	2.3	4.9	45.0
Age										
55–64 years (*n* = 684)	284	25.4	12.3	13.0	6.3	6.7	1.8	4.2	5.3	40.5
65–79 years (*n* = 1,281)	408	19.4	17.2	13.5	7.8	11.8	1.7	3.7	6.1	38.2
≥80 years (*n* = 593)	130	11.5	13.9	5.4	5.4	17.7	2.3	2.3	3.9	50.8
Race/ethnicity										
Hispanic (*n* = 236)	53	28.3	18.9	13.2	5.7	11.3	0.0	0.0	7.6	39.6
NH-Black^k^ (*n* = 359)	155	17.4	20.7	9.0	8.4	16.8	1.9	1.3	2.6	40.0
NH Other^l^ (*n* = 84)	37	27.0	18.9	13.5	5.4	8.1	5.4	8.1	10.8	43.2
NH-White (*n* = 1,870)	576	19.8	12.9	12.7	6.8	9.6	1.7	4.3	5.7	41.2
Marital status										
Living w/partner (*n* = 92)	27	14.8	0.0	14.8	0.0	18.5	0.0	0.0	3.7	51.9
Married (*n* = 1,651)	550	21.6	12.6	12.6	7.1	8.9	2.6	5.5	5.1	41.3
Never married (*n* = 96)	31	9.7	16.1	16.1	3.2	16.1	0.0	0.0	6.5	51.6
Separated/divorced (*n* = 276)	97	20.6	19.6	7.2	12.4	14.4	0.0	5.2	5.2	40.2
Widowed (*n* = 408)	113	16.8	24.8	12.4	4.4	15.0	0.88	5.3	8.0	34.5
Education										
No HS diploma (*n* = 253)	39	15.4	20.5	2.6	2.6	18.0	2.6	0.0	2.6	48.7
HS diploma/GED (*n* = 535)	132	18.2	12.1	9.9	6.1	15.2	0.76	2.3	4.6	41.7
Associates/vocational (*n* = 911)	287	20.2	19.9	11.2	7.0	13.6	2.1	3.8	3.8	39.7
Bachelors/graduate (*n* = 859)	364	21.4	11.5	14.6	7.7	6.6	1.9	4.4	7.4	40.9
Household income^m^										
<25,000 (*n* = 445)	119	15.1	17.7	10.1	5.9	16.0	2.5	0.84	6.7	45.4
25,000–49,999 (*n* = 558)	152	22.4	20.4	10.5	3.3	11.8	2.0	4.6	5.3	39.5
50,000–99,999 (*n* = 815)	255	20.0	14.1	11.0	8.2	12.6	1.2	4.3	3.9	41.2
≥100,000 (*n* = 612)	260	22.3	10.8	15.8	8.5	6.9	2.3	3.5	7.3	38.9
Resilience score (Mean)^n^										
1–3 (*n* = 30)	2	50.0	0.0	0.0	0.0	0.0	0.0	0.0	0.0	5.0
4–6 (*n* = 414)	111	21.6	16.2	5.4	8.1	15.3	1.8	1.8	1.8	45.1
7–9 (*n* = 1,290)	435	20.0	13.6	11.0	6.2	9.9	2.1	3.2	5.1	41.8
10–12 (*n* = 490)	164	20.7	16.5	17.7	8.5	10.4	1.8	6.1	10.9	34.8
Work affected by COVID-19^o^										
Not in work force (*n* = 1,474)	427	17.3	16.2	10.3	6.8	13.4	2.1	2.3	2.2	42.6
No (*n* = 467)	135	23.7	18.5	11.1	5.9	10.4	0.74	3.0	7.6	39.3
Yes (*n* = 575)	249	23.7	10.4	15.7	7.6	7.2	2.0	6.4	5.4	39.0
COVID disruption score^p^										
0 (*n* = 1,371)	422	21.3	14.0	13.0	6.6	10.0	2.4	3.1	5.5	39.6
1 (*n* = 876)	297	16.5	14.5	10.8	8.4	13.1	1.4	4.4	5.7	42.4
≥ 2 (*n* = 224)	80	25.0	18.8	11.3	5.0	8.8	1.3	3.8	2.5	42.5

*Notes*: GED = General Education Development; HS = high school; NH = non-Hispanic. The column titled “Positive Change (*N*)” (top to bottom) is the number of respondents endorsing a positive change by sociodemographic characteristics. The row titled “Overall *N*” (left to right) represents the unweighted number of respondents reporting a positive change and those identifying a specific change in the open response field. The row titled “Percent” is the percent of respondents with each specific change. Starting at row 3, column 2, all columns under the specific change themes are the percent endorsing the identified specific change. The numerator is the overall *N* (row 2), and the denominator is the total number of people specifying a change (*N* = 822).

^a^Number of respondents aged 55 years and older reporting a positive change during the pandemic. Thirty-four percent of respondents reported endorsement of a positive change during the pandemic. Categories of positive change are not mutually exclusive.

^b^Open-ended responses including keywords/phrases such as getting to know, rekindled, communication, closer, quality, time, with family, friends, relatives, and or children were categorized as having a positive change involving time.

^c^Open-ended responses, including keywords/phrases such as church, bible, pray, worship, meditation, reflection, spiritual, or God, were categorized as having a positive change involving spirituality.

^d^Open-ended responses including keywords/phrases such as health, diet, quit drinking or drinking less, quit smoking, or smoking less, weight loss, exercise, physical activity, or talk more were categorized as having a positive change involving health.

^e^Open-ended responses including keywords/phrases such as cleaning, organizing, home projects, paring down, remodeling, renovation, or redecorating were categorized as having a positive change involving the home.

^f^Open-ended responses including keywords/phrases such as social distancing, disinfecting, sanitizing, staying away from others, masking, wearing gloves, home projects, paring down, remodeling, renovation, or not leaving the house were categorized as having a positive change involving hygiene.

^g^Open-ended responses including keywords/phrases such as money, finances, saving money, spending, or income were categorized as having a positive change involving money.

^h^Open-ended responses including keywords/phrases such as social anxiety, calming, stress, balance, or slowed down were categorized as having a positive change involving stress.

^i^Open-ended responses including keywords/phrases such as hobbies, playing music, bridge, learned, sewing, knitting, quilting, painting, reading, writing, or birdwatching were categorized as having a positive change involving hobbies.

^j^Respondents reporting a positive change who did not report a specific change, and those with a specific change that did not fall within the following detailed categories were categorized as other.

^k^Survey race question did not distinguish between African American and Black.

^l^Races categorized as Other were those who responded Native American/Alaskan Native (0.74%), Asian/Pacific Islander (2.0%), and Other (7.6%).

^m^Household income was derived from unfolding brackets; those responding, “do not know/refused” were asked the specific income question were asked, “Would you say the income of your household was more or less than $50,000? Those responding less were asked, “Would you say the income of your household is more than $25,000 or less than $25,000?” and those responding more were, “Would you say the income of your household is more than $100,000 or less than $100,000?”

^n^Resilience score was calculated from the 4-item trait resilience measure. Responses were summed to create a total resilience score ranging from 0 to 12, with higher scores representing greater resilience.

^o^Respondents were asked “Has your work been affected by the COVID-19 pandemic?” “Not working” capture those who were not working when the pandemic started.

^p^The COVID disruption score was calculated summing five dichotomous items: living alone, COVID-19-related deaths among friends/family, residential relocation of self, others moving in due to the pandemic, and pandemic effect on income. The disruption score ranged from 0 to 5, with higher scores representing greater disruption.

## Discussion

We examined whether FCT and trait resilience contributed to some older adults endorsing a positive change during the pandemic. FCT posits that SES is a “fundamental cause” of differences in health, embodying resources that people can use to protect against adverse health outcomes (Link & Phelan, 1995). Educational attainment and household income, indicators of SES, were examined as contributors to a person’s ability to find something positive during the pandemic. Trait resilience encompasses attributes such as self-efficacy, critical thinking skills, and purpose, which may help individuals maintain a positive mindset, reframe a challenge, or seek social support during the hardships of the pandemic.

Our study found that educational attainment was significantly associated with an endorsement of a positive change, even adjusted for income. In contrast, income and resilience were not associated with endorsing a positive change in an adjusted model that included education. Educational attainment is a complicated variable; it is a proxy for knowledge and a complex set of related endowments; and higher educational attainment may increase a person’s sources of knowledge transfer and the ability to digest information to solve problems. Attributes of higher educational attainment may contribute to a person’s adaptive capacity (Striessnig et al., 2013), allowing for the identification and endorsement of positive changes during the pandemic. Health literacy is highly correlated with educational attainment; studies have found that patient activation (self-management, collaboration with health care providers, prevention of illness, and self-efficacy) is significantly associated with education (Eneanya et al., 2016). This correlation was expected, as health literacy also includes the ability to digest and synthesize information from multiple sources. A person’s ability to disentangle varied and contradictory information about transmission, risk reductions, and severity may improve older adults’ ability to engage in positive actions.

In addition, individuals with higher educational attainment may have been more likely to have jobs that allowed them to transition to remote or work-from-home arrangements during the pandemic. Although it was not statistically significant in the final model, a larger proportion of respondents whose work was affected by the pandemic reported positive changes compared to those who were not in the workforce or whose work was not affected. This trend could be related to people who started working remotely finding it to be advantageous. More education could also be associated with greater capacity (access, knowledge) to use the technologies needed for virtual interactions; this may have contributed to a strong association between education and the “Social Relationship” category of positive changes. Higher educational attainment manifests in diverse resources, such as money, knowledge, or social connections as described in the FCT.

Surprisingly, resilience was not significantly associated with the endorsement of positive changes, after adjusting for education and income. Certain aspects of resilience, such as positive reframing, could be beneficial during the pandemic, for example, helping some respondents reframe mandated social distancing as an opportunity to spend more quality time with family or fix up the home. Resilience is not independent of FCT; higher resilience may be related to greater ability to access flexible resources, including money, knowledge/education, power, prestige, or social connections, as posited by FCT. At the same time, income, education, and social connections may enhance an individual’s ability to develop and maintain a sense of resilience based on their objective and subjective assessments of resources that they can bring to bear in the face of challenges. However, just as the distribution of resources is influenced by age, gender, income, family and social relationships, and health, so may resilience be (Araki, 2022; Wister et al., 2016).

Black older adults had twice the odds of endorsing any positive change despite being disproportionately affected by lower incomes and less educational attainment. One explanation may be the effect of repeated exposure to stressors over time, which increases a person’s adaptive capability (Shing et al., 2016). Due to historical and cultural inheritance and systemic racism, NH Black older adults may have developed strategies as part of their adaptive ability in response to continual exposure to racism. The pandemic may have seemed minor as a challenge to be managed. Black older adults had higher occurrences of positive changes related to spirituality, which may have aided in their adaptability (Mouzon, 2022). Also stemming from historical and cultural inheritance, the findings may be biased by survivorship because older Black adults may be a relatively more elite group in their physiological, psychological, and social functioning compared to their White counterparts (Jackson et al., 1982). Additionally, there were higher occurrences of positive changes related to hygiene among respondents who were Black, low-income, or had lower educational attainment. These changes may have been attempts among these subgroups to compensate for an inability to reduce interactions (i.e., in-person employment or dependency on public transit), or it may be that more educated and affluent respondents did not consider their improved hygiene to be a positive change but simply followed recommendations.

### Strengths and Limitations

A strength of our study is that we have data at two time points. We linked the NSHAP respondents’ data collected in the first year of the pandemic to their earlier 2015–2016 responses. We captured prior measurements of educational attainment, household income, and resilience not influenced by the pandemic, whereas many other studies looking at “prepandemic predictors” actually use data collected earlier in the pandemic when these factors may already have been influenced by the upheaval of the pandemic (Pieh et al., 2020; Sibley et al., 2020).

It is important to note that our resilience scale was developed by drawing items from extant resilience scales and showed good discriminative and convergent validity (Hawkley et al., 2021). However, our scale has yet to be tested for generalizability across racial-ethnic subgroups, a limitation of all extant resilience scales. Consequently, our results may under- or overestimate group differences in resilience as operationalized here. Future research should explore whether resilience is equivalently conceived and operationalized across racial-ethnic groups.

Asking about positive change during the pandemic rather than by recall after the crisis phase of the pandemic is advantageous in that responses are temporally proximal to respondents’ experiences. However, the specific changes were captured with an open-ended question, and spontaneous recall may have led to respondents forgetting some changes. A prompt (i.e., a choice list) may have assisted in remembering positive changes, but it may have also been more likely to elicit socially desirable responses. The investigators’ preconceptions about what would be considered a positive change did not limit their responses. Not constraining respondents’ responses revealed interesting group differences in spontaneously generated examples of positive change. There is inevitably some subjectivity though in categorizing unconstrained responses.

There is value in understanding the circumstances that foster the ability to recognize positive changes in the face of challenges. FCT holds that SES is one beneficial circumstance. This theory most often operationalizes SES with indicators of educational attainment and household income, but these indicators may be inadequate to understand other ways in which COVID- and other stress-related changes are experienced. For instance, although Black Americans are underrepresented in those with higher household income or higher education, they may have access to other assets that may compensate for low income and education and enhance their ability to experience and identify positive changes. Research investigating a range of assets could provide valuable insights into how to promote access to resources that support wellness in marginalized communities.

## Conclusion

This work extends the application of FCT to a health-related outcome that is not health per se but rather the ability to navigate a complicated health-related life challenge. Flexible resources affect one’s ability to persevere during challenging times and add to the literature on behavior and adaption amid the pandemic’s physical, financial, psychological, and health challenges. Our study has found that higher educational attainment is strongly associated with greater odds of endorsing a positive change during the pandemic, among older adults. This relationship may be due to an increased ability to synthesize information from multiple sources and be less overwhelmed by fragmented and contradictory information. Being less overwhelmed may enable one to identify and act on what was most salient to their wellness during the pandemic. While resilience was not explanatory of the endorsement of positive changes during the pandemic, how FCT and resilience relate to each other needs further exploration. Research is needed to understand the short- and long-term effects of the experience and identification of positive changes on long-term mental and physical health and well-being. In addition, besides SES, other fundamental causes (e.g., social connection and social support) need to be included in research to better understand their role in supporting positive outcomes, whether directly or indirectly through support of personal resilience. Finally, further examination of the relationship between fundamental causes and resilience is needed, with particular attention to the possibility that the association differs by age, sex, and race/ethnicity.

## Supplementary Material

igad058_suppl_Supplementary_Materials
