# Supplementary material for: Did Resilience and Socioeconomic Status Predict Older Adults’ Finding a Silver Lining in COVID?
Source: Innov Aging. 2023 Jun 21;7(6):igad058. doi: 10.1093/geroni/igad058 (PMC10500972; doi:10.1093/geroni/igad058)
Supplement: igad058_suppl_Supplementary_Materials [file igad058_suppl_supplementary_materials.docx]

**Supplementary Table 1. Correlations of Resilience Score, Educational Attainment and Household Income.**

| Variable | Resilience^1^ | Education^2^ | Income^3^ |
| --- | --- | --- | --- |
| Resilience^1^ | 1.0 |  |  |
| Education^2^ | 0.10 | 1.0 |  |
| Income^3^ | 0.16 | 0.48 | 1.0 |

^1^Resilience score was calculated from the 4-item trait resilience measure. Responses were summed to create a total resilience score ranging from 0 to 12, with higher scores representing greater resilience.

^2^Educational attainment was categorized into four levels: non-high school (HS) graduates, HS graduates/GED, associate degree/vocational degree, and bachelors/graduate degree.

^3^Household income was derived from unfolding brackets; those responding, "don't know/refused" were asked the specific income question: "Would you say the income of your household was more or less than $50,000? Those responding less were "Would you say the income of your household is more than $25,000 or less than $25,000?" and those responding more were "Would you say the income of your household is more than $100,000 or less than $100,000?"

**Supplementary Table 2.** **Sociodemographic Characteristics Overall and Based on Endorsement of Positive Change^a^ and Type of Specification (Weighted)**

| **Variable** | **Overall** | **No change** | **Positive Change** | **Positive change -unspecified** | **Positive change -specified^1^** |
| --- | --- | --- | --- | --- | --- |
| **Gender** |  |  |  |  |  |
| Female | 55.2 | 51.4 | 61.0 | 58.22 | 64.2 |
| Male | 44.8 | 48.6 | 39.0 | 41.78 | 35.8 |
| **Age** |  |  |  |  |  |
| 55-64 years | 42.5 | 35.4 | 50.9 | 51.1 | 49.8 |
| 65-79 years | 44.6 | 45.8 | 41.2 | 39.0 | 43.1 |
| ≥ 80 years | 12.9 | 14.8 | 8.0 | 9.9 | 7.1 |
| **Race/Ethnicity** |  |  |  |  |  |
| Hispanic | 6.7 | 7.2 | 5.6 | 4.4 | 6.5 |
| Non-Hispanic-Black ^b^ | 10.4 | 7.5 | 14.1 | 14.5 | 14.1 |
| Non-Hispanic-Other ^c^ | 3.5 | 2.8 | 4.5 | 5.2 | 4.0 |
| Non-Hispanic-White | 79.3 | 82.4 | 75.6 | 75.9 | 75.4 |
| **Marital Status** |  |  |  |  |  |
| Living with partner | 4.1 | 4.2 | 3.3 | 3.7 | 3.0 |
| Married | 65.9 | 64.6 | 70.2 | 69.1 | 69.2 |
| Never Married | 4.9 | 4.8 | 4.3 | 6.0 | 3.7 |
| Separated/Divorced | 13.3 | 14.0 | 12.6 | 12.7 | 12.6 |
| Widowed | 11.8 | 12.4 | 9.5 | 8.5 | 11.4 |
| **Educational Attainment** |  |  |  |  |  |
| No HS diploma | 7.5 | 9.0 | 3.4 | 4.0 | 3.5 |
| HS Diploma/GED | 21.2 | 24.6 | 14.8 | 15.4 | 14.4 |
| Associates/ Vocational | 36.6 | 37.2 | 34.2 | 32.2 | 36.5 |
| Bachelors/Graduate | 34.7 | 29.2 | 47.7 | 48.4 | 45.6 |
| **Household Income** ^d^ |  |  |  |  |  |
| < 25,000 | 17.2 | 18.4 | 13.3 | 14.6 | 14.5 |
| 25,000-49,999 | 20.6 | 21.2 | 18.2 | 19.4 | 18.0 |
| 50,000-99,999 | 31.3 | 33.9 | 28.5 | 26.9 | 28.4 |
| >=100,000 | 31.0 | 26.4 | 40.0 | 39.1 | 39.1 |
| **Resilience Score (Mean)** ^e^ |  |  |  |  |  |
| 1-3 | 1.6 | 2.2 | 0.40 | 0.66 | 0.21 |
| 4-6 | 16.9 | 18.3 | 14.9 | 16.7 | 13.3 |
| 7-9 | 60.4 | 58.4 | 63.7 | 64.7 | 63.5 |
| 10-12 | 21.1 | 21.2 | 21.1 | 17.9 | 23.0 |
| **Work affected by COVID-19 ^f^** |  |  |  |  |  |
| Not in work force | 48.2 | 52.2 | 40.9 | 41.8 | 41.4 |
| No | 20.3 | 20.7 | 18.3 | 17.2 | 18.8 |
| Yes | 31.5 | 27.1 | 40.9 | 41.0 | 39.8 |
| **COVID Disruption Score ^g^** |  |  |  |  |  |
| 0 | 31.0 | 32.9 | 26.9 | 27.9 | 26.5 |
| 1 | 59.6 | 59.5 | 60.4 | 56.8 | 62.8 |
| ≥ 2 | 9.4 | 7.6 | 12.7 | 15.3 | 10.7 |

Notes. HS = high school; GED = General Education Development. 6% of those specifying a positive change reported 3 or more positive changes.

^a^ Of the respondents asked, "Has the COVID-19 pandemic led to any positive change in your life" 162 are missing responses.

^b^ Survey race question did not distinguish between African American and Black.

^c^ Races categorized as Other were those who responded Native American/Alaskan Native (0.74%), Asian/Pacific Islander (2.0%) and Other (7.6%).

^d^ Household income was derived from unfolding brackets, "do not know/refused" were asked the specific income question, "Would you say the income of your household was more or less than $50,000?” Those responding less than were asked, "Would you say the income of your household is more than $25,000 or less than $25,000?" and those responding more than were asked, "Would you say the income of your household is more than $100,000 or less than $100,000?"

^e^ Resilience score was calculated from the 4-item trait resilience measure. Responses were summed to create a total resilience score ranging from 0 to 12, with higher scores representing greater resilience.

^f^ Respondents were asked “Has your work been affected by the COVID-19 pandemic?” “Not working” capture those who were not working when the pandemic started.

^g^ The COVID disruption score was calculated summing five dichotomous items: living alone, COVID-19-related death among friends/family, residential relocation of self, others moving in due to the pandemic, and pandemic effect on income. The disruption score ranged from 0 to 5, with higher scores representing greater disruption.
